# What helps people to reduce or stop self-harm? A systematic review and meta-synthesis of first-hand accounts

**DOI:** 10.1093/pubmed/fdac022

**Published:** 2022-02-24

**Authors:** Cathy A Brennan, Helen Crosby, Cara Sass, Kate L Farley, Louise D Bryant, Rocio Rodriquez-Lopez, Daniel Romeu, Elizabeth Mitchell, Allan O House, Else Guthrie

**Affiliations:** Leeds Institute of Health Sciences, School of Medicine, University of Leeds, LS2 9LJ Leeds, UK; Leeds Institute of Health Sciences, School of Medicine, University of Leeds, LS2 9LJ Leeds, UK; School of Psychology and Therapeutic Studies, Leeds Trinity University, LS18 5HD Leeds, UK; Leeds Institute of Health Sciences, School of Medicine, University of Leeds, LS2 9LJ Leeds, UK; Leeds Institute of Health Sciences, School of Medicine, University of Leeds, LS2 9LJ Leeds, UK; Leeds Institute of Health Sciences, School of Medicine, University of Leeds, LS2 9LJ Leeds, UK; Leeds Institute of Health Sciences, School of Medicine, University of Leeds, LS2 9LJ Leeds, UK; Leeds Institute of Health Sciences, School of Medicine, University of Leeds, LS2 9LJ Leeds, UK; Leeds and York Partnership Foundation Trust, LS15 8ZB Leeds, UK; School of Psychology, University of Leeds, LS2 9JT Leeds, UK; Leeds Institute of Health Sciences, School of Medicine, University of Leeds, LS2 9LJ Leeds, UK; Leeds Institute of Health Sciences, School of Medicine, University of Leeds, LS2 9LJ Leeds, UK

**Keywords:** cessation, meta-synthesis, self-harm, self-management, systematic review

## Abstract

**Background:**

Self-harm is an important public health problem but therapeutic interventions, particularly for people who have a history of multiple repetition, are not always taken up or effective when they are. The aim of this review is to explore first-hand accounts of what helps outside therapy and identify actions and processes, which can support the reduction or cessation of self-harm.

**Methods:**

A systematic review and thematic meta-synthesis of the first-person accounts of what has helped to reduce or stop self-harm reported in primary studies.

**Results:**

The meta-synthesis combined 546 participant excerpts from 56 studies. Two over-arching themes were identified: (i) *breaking the chain* incorporated actions taken to break the link between a person’s current psychological or social state and the act of self-harm and (ii) *building a new foundation for change* captured actions over the longer-term, focusing on practical changes in relationships and in a person’s way of life, such as work or living arrangements.

**Conclusions:**

The results emphasize the importance of interpersonal change in reducing or stopping self-harm. While interpersonal factors are acknowledged as important reasons behind self-harm, they are often under-represented in self-management advice and therapeutic interventions that focus on individual psychopathology.

## Introduction

Lifetime prevalence of self-harm is about 7% in UK.^1^ It is associated with a range of health problems, poor quality of life and an increased risk of suicide.^2^^,^^3^ There is a lack of evidence for therapeutic effectiveness after self-harm and especially repeated self-harm.^4^ Reasons for self-harm are diverse and there is a need for interventions that go beyond managing negative emotions.^5^^,^^6^ Supported self-management is likely to be an important part of such interventions.^7^ A review that examines what individuals themselves report to have been helpful is important for developing more effective self-management resources, either stand alone or part of person-centred therapies.

For this review we adopted the National Institute for Health and Care Excellence definition of self-harm: ‘*any act of self-poisoning or self-injury carried out by a person, irrespective of their motivation*’ pg. 6,^8^ which encompasses all non-suicidal self-harm and non-fatal suicidal behaviours, allowing a comprehensive view without making assumptions about the presence or absence of intent to die.^9^^,^^10^

The objectives for the review are: (i) to identify actions reported as being associated with reduction or cessation of self-harm and (ii) to identify mechanisms that might explain benefits, highlighting actions that can be a part of supported self-management.^8^

## Methods

### Study identification

An information specialist with expertise in literature search methods developed an exhaustive search strategy^11^ that included terms to identify self-harm and suicidal behavior combined with terms to identify reduction, cessation or self-management (full strategy for Medline is in the Supplementary Material). A qualitative study design filter excluded studies that were unlikely to contain first-person accounts. The strategy was adapted for each database using free-text and indexing terms.

The following databases were searched up to August 2019: MEDLINE, Embase, PsycINFO, Web of Science: Citation Index and Conference Proceedings, CINAHL, Epistemonikos, Cochrane Database of Systematic Reviews and Cochrane Central Register of Controlled Trials.

Additional studies were identified from reference lists of review articles identified from the database searches and of included studies. Citation tracking used Google Scholar and Web of Science.

### Study selection

**Fig. 1:**
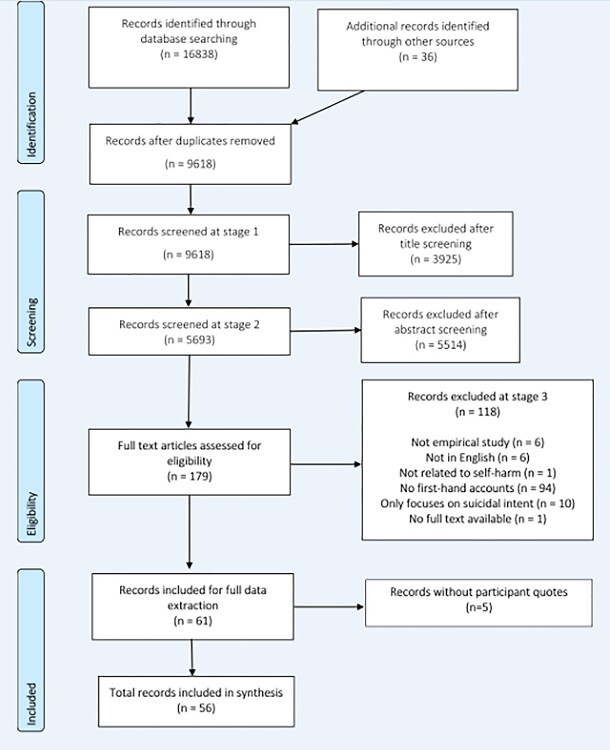
Flow of studies through selection process.

Eligible studies were identified using a three-stage process. First, titles of articles were screened by one reviewer. Excluded studies were screened by a second reviewer to ensure consistency. Second, titles and abstracts of articles from the resulting list of potential articles were independently screened by two reviewers against the inclusion criteria (Table 1). Third, full manuscripts of articles selected in stage 2 were assessed by two reviewers independently. Discrepancies were resolved through consensus. The selection process is summarized in a flow chart (Figure 1).

### Data extraction and quality assessment

Data extraction and quality assessment were performed independently by two reviewers. Quality was assessed using adapted versions of COREQ (COnsolidated criteria for REporting Qualitative research) or STROBE (Strengthening the Reporting of Observational studies in Epidemiology) depending on study design. No studies were excluded on the basis of quality scores.

The following information was tabulated from the included articles: study identifiers (first author, publication year and language), aims, method of data collection, study definition of self-harm, participants (sample size, age range [or mean and standard deviation if range not reported], gender, other reported demographic characteristics, method of harm), research setting and country and findings (first-hand accounts of how people have reduced or stopped self-harm, which included verbatim quotations from participants or qualitative responses on questionnaires.).

**Table 1:** Criteria for inclusion and exclusion

Inclusion criteria	Exclusion criteria
• Studies that report first-hand accounts associated with reduction or cessation of self-harm from people who have self-harmed. • Studies of individuals of any age, gender or ethnicity. • Studies of individuals with or without co-occurring psychiatric disorders. • Studies across all motives (non-suicidal or suicidal) and methods (poisoning or self-injury) of self-harm.	• Studies that focus solely on suicidal intentions. • Studies that report factors associated with a reduction or cessation of self-harm but not first-hand accounts. • Studies that report only second-hand accounts of how people have reduced or stopped self-harm, e.g. healthcare professionals’ views towards self-harm reduction or cessation. • Studies published in languages other than English.

### Data synthesis

We used a form of thematic synthesis^12^^,^^13^ using the first-person quotations extracted from each study as our data. The synthesis involved two phases:

#### Phase 1: Developing the thematic framework

First, each quotation or questionnaire response was coded according to its meaning and content. Second, codes of similar meanings were grouped into descriptive themes. The last stage involved generation of analytical themes to ‘go beyond’ the content of the original data,^12^ offering new conceptualizations of context and relationships between the identified actions and underlying mechanisms. This was a collaborative process with each piece of data discussed by team members and all potential codes and meanings captured. The grouping into themes was an iterative process that continued until consensus was reached. The analytic team included topic experts (EG, AH), social scientists with qualitative methods skills (CB, HC, KF, LB) and an expert in literature searching (RL).

#### Phase 2: Refining the thematic framework

In Phase 2, a reference group of 12 people with experience of self-harm and three members of the review team reviewed and refined the thematic structure developed in Phase 1. Once the thematic structure had been refined, theme labels and definitions were generated.

## Results

The search strategy found 9618 studies after duplicates had been removed. Of these, 56 met the inclusion criteria. We could not access the full text of one potentially eligible study. The quality of the studies was generally high with 69% recorded as good quality. A list of the included studies is given in the Supplementary Materials.

Most studies came from Europe (21) and North America (18), with five studies based in Asia, two in New Zealand and five studies with unspecified samples. In total there were 2837 participants across 56 studies, minimum sample size 4, maximum 836. Thirteen studies included young people only, 21 working age adults and the rest a mixed age sample. Participants were recruited from clinical services in 17 of the studies and two studies were in prison populations.

**Fig. 2:**
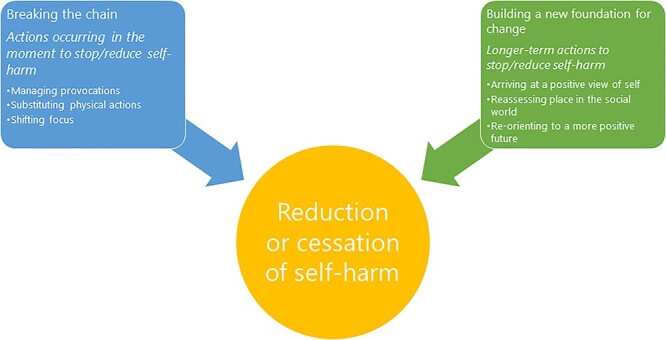
Thematic map.

A total of 546 individual quotations were extracted from the included studies. The synthesis gave two meta-themes: *breaking the chain* and *building a new foundation for change*. Figure 2 shows the thematic map and Table 2 shows the number of articles and quotes contributing to the sub-themes.

### Meta-theme one: Breaking the chain

Actions taken in the moment to break the link between a person’s current psychological or social state and the act of self-harm involved: *shifting focus*, *substituting physical actions, managing provocations* and *critical appraisal of self-harm.*

Shifting focus encapsulated the strategies used to make thoughts of self-harm less intrusive. Some used meditative practices, finding quiet places to reflect and experience a sense of connection to the self. In contrast, many described the importance of keeping occupied (in enjoyable hobbies or mundane tasks for example) and avoiding quiet environments that could allow negative thoughts to gain prominence.

Some strategies involved creative activities for self-expression or to communicate feelings to others. The notion of a ‘creative release’ involved channelling feelings into a creatively fulfilling artefact. Some individuals described a further purpose—artistic outlays such as writing or drawing formed a tangible record of self-harm or a form of communication with the self and enabled people to reflect on their emotions and urges to self-harm.

### Substituting physical actions involved direct replacement of self-harm with another act

Accounts of substitution acknowledged the destructive nature of self-harm and most explored acts of self-care and pleasurable activities such as enjoying a meal or treating oneself to clothes. However, not all substitutes were healthy. In one study, an individual substituted self-harm with smoking, explaining ‘*you don’t die straight away from smoking*’ pg. 372.^14^ One study suggested self-administered acupuncture^15^ as a substitute.

*Managing provocations* captured strategies that recognized and avoided events and social interactions (especially with key people) that were known to lead to thoughts of self-harm. Many individuals used the term ‘triggering’ to describe precipitating experiences. For some this meant exercising care when online, although there was not consensus about what content may be a trigger: some accounts described avoiding online images of self-harm, others described looking at such images as a substitute for the act itself.

Some participants identified their past use of alcohol as a way of coping with stress, while acknowledging that this limited their capacity to make decisions and manage their health. Participants noted their increased likelihood to harm themselves if they were intoxicated, sobriety reflected a choice to gain greater control.

Critical appraisal of self-harm described a shift toward more reflective thinking about the determinants of self-harm and the differences between its perceived and actual outcomes. Some self-harm escalated to a greater degree than anticipated, or the healing process for injuries was complicated by persistent bleeding or infection—leading to a re-evaluation of the worth of self-injury with its downside of incurring ‘ugly’ physical scars. For some reappraisal involved a realization of the futility of the act in that it gave no rational benefit.

**Table 2:** Breakdown of saturation of themes

Meta-theme	Sub-theme	No. articles	No. quotes
Breaking the chain	Shifting focus	19	31
	Substituting physical actions	13	16
	Managing provocations	7	9
	Critical appraisal of self-harm	5	15
Building a new foundation for change	Arriving at a positive view of self	35	98
	Reassessing place in the social world	32	91
			
	Re-orienting to a more positive future	16	28

### Meta-theme two: Building a new foundation for change

The second meta-theme referred to longer-term actions that strengthened the separation between self-harm and a person’s way of life: *arriving at a positive view of self*, *reassessing place in the social world* and *re-orientating to a more positive future*.

Arriving at a positive view of self describes reaching a turning point at which respondents saw themselves as worthy of help and support. For many this was about understanding their own vulnerability to mental health difficulties and being open to seeking help when needed. Some participants reported that validation from others had helped—another person’s efforts to understand their situation, having someone who believed in them or knowing that someone would stand by them unconditionally.

Some participants described the benefit of being able to reveal their true identities to others. This was particularly salient in accounts from a study describing distress at attempts to conceal sexuality.^16^

Numerous accounts referred to individuals finding the self-determination to stop, making efforts to refocus their energies, to achieve mastery over self-harm. These accounts were not directly linked to a cause or event of significance, but a gradual shift in beliefs about self-harm. Change needed to focus on the idea that through gradual change it is possible to gain control.

Other accounts indicated a more passive process, for example describing a gradual diminishing of impulses. For some this was associated with the progression through life stages, such as adolescence or getting married and starting a family.

Reassessing place in the social world describes the impact of social factors and in particular how changes in personal relationships were important drivers for cessation of self-harm. An important aspect to this was acting on a need to break with damaging relationships. In some cases individuals were not prepared to, or could not, break social connections that were detrimental to their wellbeing. In these situations an alternative was to redefine these relationships by consciously choosing not to focus on upsetting past events or to focus solely on what was positive in the relationships.

In some accounts, a physical change in circumstances was necessary by for example moving house or neighbourhood*.* For others there was a need for a temporary move in times of crisis to a place away from damaging relationships, including time in hospital.

Re-assessing one’s place in the social world could also include appraisal of those people to whom one related closely. Participants talked about the particular importance of relating to others with a shared experience of self-harm—through reading blogs, sharing stories online or joining peer-led support groups. Individuals stressed the importance of non-judgmental others.

A further aspect of re-assessing one’s place in the social world was accounts of reducing or stopping self-harm motivated by a sense of one’s responsibility for others. Membership of support groups helped some, because they sensed others were paying attention to their actions and feared disappointing them. Focusing on positive experiences, such as raising children, grandchildren or loved pets could be a driver for change. In many cases the motivation was internal—worry about the impact of self-harm or how it might be perceived by others. Others mentioned having made verbal or written contracts with others to create an additional barrier to self-harm.

Re-orienting to a more positive future captures the factors that allowed participants to begin to move on with their lives. An important precursor for cessation was reaching an understanding that change was possible but that it might take time, allowing participants to take a step back from a sense of captivity in the immediate.

Individuals described how they began to think positively about life developments and actively sought to find hope for the future: *‘finding the goodness in life rather than the goodness in death’* pg 466*.*^17^ Some found hope through focusing on new goals, such as working toward qualifications or finding work that gave a sense of personal fulfilment. Others talked about visualizing their recovery and focusing on a time in the future.

Harnessing hope required effort. Some participants described how faith, spirituality and religion helped, giving feelings of comfort or helping individuals to feel empowered to change. Conversely, some first-hand accounts indicated a moral opposition to suicide and a fear of not being able to join family members in the afterlife. What was important in all these accounts was a sense that participants could see something to work towards.

## Discussion

### Main finding of this study

This meta-synthesis identified two broad processes involved in reducing self-harm.

*Breaking the chain* encompassed strategies to manage immediate thoughts and feelings while *building a new foundation for change* encompassed strategies that included longer-term mechanisms. This suggests that work to reduce self-harm requires some actions not only for immediate effects but also action for longer term change in a person’s wider social circumstances.

### What is already known on this topic

These results regarding short-term strategies for managing self-harm are consistent with advice that can be found online.^18^^,^^19^

### What this study adds

Our review has highlighted differences in emphasis from much self-management advice.

First, in our review the theme of substitution as a strategy overwhelmingly referred to replacement with more positive experiences such as acts of self-care. Advice on substitution strategies often mentions activities such as flicking elastic bands against wrists, rubbing ice cubes on skin or dropping hot sauce on tongues as ‘safer’ ways to inflict pain. Such techniques have little evidence for effectiveness,^20^ and recent research has confirmed the suggestion from our review that it is unusual for people to use painful substitutes.^21^

Second, managing provocations was highlighted as an important strategy in breaking the chain. This included recognizing and avoiding triggers and awareness of contributing factors such as alcohol. Much of the discourse on managing ‘triggers’ is dominated by discussion of restricting access to text or images about self-harm, especially online, but while looking at images of self-harm or reading accounts of self-harm may contribute to urges to self-harm, for some looking at images online was a helpful substitute action.

More importantly, the provocations identified in our review were mainly from interpersonal encounters—unsurprisingly since most accounts of precipitating factors for self-harm cite arguments or unhelpful encounters with others.^22^ Reappraising and disrupting unhelpful interpersonal relationships were also prominent strategies in the second meta-theme in our review—building new foundations for change.

This finding illuminates an imbalance in the dominant discourse on repeated self-harm—which locates the problem in individual psychopathology rather than as an understandable reaction to interpersonal tensions. What is noteworthy is the importance of establishing lasting change in social and interpersonal circumstances and not just an emphasis on changing feelings about self. What is underrepresented in the professional discourse is accounts that highlight the importance of structural changes in circumstances—leaving toxic relationships, moving out of family homes, finding purpose through employment were all evident as important strategies for achieving lasting change.

### Limitations of this study

We used wide inclusion and minimal exclusion criteria and a broad definition of self-harm, and the literature we found does not allow differentiation between resources to help self-harm from those that might help people who wish more specifically to end their lives, nor on actions that might stop self-harm completely as opposed to lead to gradual reduction or mitigation. There was no date restriction but all included studies had been published in the last 20 years so were felt to be relevant for inclusion. Priority was given to first-hand accounts, which meant that potentially helpful elements of social media use were not comprehensively identified because of the nature of that literature. The exclusion of articles without an English language version means that different cultural influences may not be represented. We were constrained by the analysis and thematic frameworks of the primary studies in that we can only synthesis quotes that have been selected for inclusion in these papers. While we kept the context of the quotations to aid analysis, we synthesized the data without the context of the study it came from, so our analysis did not involve translation of concepts across studies. The included studies were heterogeneous and we did not seek to identify for whom and in what contexts each of the mentioned strategies might be effective.

### Implications

Our findings indicate that advice on avoiding triggers or short-term provocations to self-harm needs to take more account of how often they involve interpersonal interactions. Approaches to identifying, stepping away from or disrupting problematic social situations are used for example in anger management techniques^23^ and could be more widely employed in advice in the setting of self-harm.

The current debate on the influence of social media is moving from a preoccupation with restriction of access to self-harm content, to a more balanced understanding of the benefits as well as potential harm in online content. If some people find it helpful to access self-harm content via social media, then our review suggests that attention is needed to enable it as a resource not only for those seeking short-term solutions but also for those who need support on a longer trajectory towards emotional and personal change.

Our findings about making changes to the social and interpersonal world suggest this is an important area to develop in future interventions. Here, however, deciding on the practical implications is more problematic. The particulars will vary by individual, and it is inadvisable to make blanket suggestions about important life changes as part of self-management advice. At the least, our findings point to a need to broaden the remit from a current predominant focus on the personal, to include a more interpersonal and social dimension to supported self-management—suggesting a move away from the predominance of clinical interventions to those with a more societal focus.

## Supplementary Material

Supplementary_file_list_of_included_studies_fdac022

Supplementary_file_Medline_search_strategy_fdac022

## Notes

**Cathy A. Brennan,** Associate Professor of Psychological and Social Medicine

**H. Crosby,** Research Fellow Lecturer in Pschology

**C. Sass,** Research Fellow

**K. Farley,** Senior Research Fellow

**L.D. Bryant,** Professor of Psychological and Social Medicine

**R. Rodriguez Lopez,** Information Specialist

**D. Romeu,** Academic Clinical Fellow in Psychiatry

**E. Mitchell,** Student and Laidlaw Scholar

**A.O. House,** Emeritus Professor of Liaison Psyhicatry

**E. Guthrie,** Professor of Psychiatry
