# Supplementary material for: What helps people to reduce or stop self-harm? A systematic review and meta-synthesis of first-hand accounts
Source: J Public Health (Oxf). 2022 Feb 24;45(1):154–61. doi: 10.1093/pubmed/fdac022 (PMC10017083; doi:10.1093/pubmed/fdac022)
Supplement: Supplementary_file_list_of_included_studies_fdac022 [file supplementary_file_list_of_included_studies_fdac022.docx]

**Supplementary file: list of included studies**

BAKER, D. & FORTUNE, S. 2008. Understanding self-harm and suicide websites: a qualitative interview study of young adult website users. *Crisis: Journal of Crisis Intervention & Suicide,* 29**,** 118-22.

BAZRAFSHAN, M. R., JAHANGIR, F. & SHOKRPOUR, N. 2017. What protects adolescents from suicidal attempt: A qualitative study. *Shiraz E Medical Journal,* 18 (9) (no pagination).

BENNETT, S., COGGAN, C. & ADAMS, P. 2002. Young People's Pathways to Well-being Following a Suicide Attempt. *International Journal of Mental Health Promotion,* 4**,** 25-32.

BERGMANS, Y., GORDON, E. & EYNAN, R. 2017. Surviving moment to moment: The experience of living in a state of ambivalence for those with recurrent suicide attempts. *Psychology and Psychotherapy,* 90**,** 633-648.

BERGMANS, Y., LANGLEY, J., LINKS, P. & LAVERY, J. V. 2009. The perspectives of young adults on recovery from repeated suicide-related behavior. *Crisis: Journal of Crisis Intervention & Suicide,* 30**,** 120-7.

BIONG, S., KARLSSON, B. & SVENSSON, T. 2008. Metaphors of a shifting sense of self in men recovering from substance abuse and suicidal behavior. *Journal of Psychosocial Nursing & Mental Health Services,* 46**,** 35-41.

BOSTIK, K. E. & EVERALL, R. D. 2007. Healing from suicide: Adolescent perceptions of attachment relationships. *British Journal of Guidance & Counselling,* 35**,** 79-96.

BOYCE, M., MUNN-GIDDINGS, C. & SECKER, J. 2018. “‘It is a safe space’: self-harm self-help groups”. *Mental Health Review Journal,* 23**,** 54-63.

BUSER, T. J., PITCHKO, A. & BUSER, J. K. 2014. Naturalistic Recovery From Nonsuicidal Self-Injury: A Phenomenological Inquiry. *Journal of Counseling & Development,* 92**,** 438-446.

CHAN, K., KIRKPATRICK, H. & BRASCH, J. 2017. The Reasons to Go On Living Project: Stories of recovery after a suicide attempt. *Qualitative Research in Psychology,* 14**,** 350-373.

CHI, M. T., LONG, A., JEANG, S. R., KU, Y. C., LU, T. & SUN, F. K. 2014. Healing and recovering after a suicide attempt: a grounded theory study. *Journal of Clinical Nursing,* 23**,** 1751-9.

CORCORAN, J., MEWSE, A. & BABIKER, G. 2007. The role of women's self-injury support-groups: a grounded theory. *Journal of Community & Applied Social Psychology,* 17**,** 35-52.

COULSON, N. S., BULLOCK, E. & RODHAM, K. 2017. Exploring the Therapeutic Affordances of Self-Harm Online Support Communities: An Online Survey of Members. *JMIR Mental Health,* 4**,** e44.

CRONA, L., STENMARKER, M., OJEHAGEN, A., HALLBERG, U. & BRADVIK, L. 2017. Taking care of oneself by regaining control - a key to continue living four to five decades after a suicide attempt in severe depression. *BMC Psychiatry,* 17**,** 69.

DAVIES, S., BELL, D., IRVINE, F. & TRANTER, R. 2011. Self-administered acupuncture as an alternative to deliberate self-harm: a feasibility study. *Journal of Personality Disorders,* 25**,** 741-54.

EVERALL, R. D., ALTROWS, K. & PAULSON, B. L. 2006. Creating a Future: A Study of Resilience in Suicidal Female Adolescents. *Journal of Counseling & Development,* 84**,** 461-471.

GELINAS, B. L. & WRIGHT, K. D. 2013. The cessation of deliberate self-harm in a university sample: the reasons, barriers, and strategies involved. *Archives of Suicide Research,* 17**,** 373-86.

GILZEAN, T. 2011. Communicating chaos, regaining control: the implications for social work of writing about self-injury. *Journal of Social Work Practice,* 25**,** 31-46.

GRIST, R., PORTER, J. & STALLARD, P. 2018. Acceptability, Use, and Safety of a Mobile Phone App (BlueIce) for Young People Who Self-Harm: Qualitative Study of Service Users' Experience. *JMIR Mental Health,* 5**,** e16.

HABERSTROH, S. & MOYER, M. 2012. Exploring an Online Self-Injury Support Group: Perspectives From Group Members. *Journal for Specialists in Group Work,* 37**,** 113-132.

HAN, C. S. & OLIFFE, J. L. 2015. Korean-Canadian immigrants' help-seeking and self-management of suicidal behaviours. *Canadian Journal of Community Mental Health,* 34**,** 17-30.

HAN, H. P., CHOU, C. H., LIU, I. C., RONG, J. R. & SHIAU, S. 2014. New start: the life experiences of recovering suicidal adolescents. *European Scientific Journal,* 10**,** 88-101.

HARRIS, I. M. & ROBERTS, L. M. 2013. Exploring the use and effects of deliberate self-harm websites: an Internet-based study. *Journal of Medical Internet Research,* 15**,** e285.

HOLLIDAY, R., BRENNAN, C. & COTTRELL, D. 2018. Understanding Adolescents’ Experiences of Self-Harm: Secondary Analysis of Family Therapy Sessions from the SHIFT Trial. *Archives of Suicide Research***,** 1-14.

HOLM, A. L. & SEVERINSSON, E. 2010. Desire to survive emotional pain related to self-harm: A Norwegian hermeneutic study. *Nursing and Health Sciences,* 12**,** 52-57.

HOLM, A. L. & SEVERINSSON, E. 2011. Struggling to recover by changing suicidal behaviour: narratives from women with borderline personality disorder. *International Journal of Mental Health Nursing,* 20**,** 165-73.

HOOVER, M. & PAULSON, B. 1999. Suicidal no longer. *Canadian Journal of Counselling,* 33**,** 227-245.

INCKLE, K. 2010. At the cutting edge: creative and holistic responses to self-injury. *Creative Nursing,* 16**,** 160-5.

KEYVANARA, M., MOUSAVI, S. G., MALEKIAN, A. & KIANPOUR, M. 2010. Suicide prevention: The experiences of recurrent suicide attempters (A phenomenological study). *Iranian Journal of Psychiatry and Behavioral Sciences,* 4**,** 4-12.

KOOL, N., VAN MEIJEL, B. & BOSMAN, M. 2009. Behavioral change in patients with severe self-injurious behavior: A patient's perspective. *Archives of Psychiatric Nursing,* 23**,** 25-31.

LEWIS, S. P. & MICHAL, N. J. 2016. Start, stop, and continue: Preliminary insight into the appeal of self-injury e-communities. *Journal of Health Psychology,* 21**,** 250-60.

LONG, M., MANKTELOW, R. & TRACEY, A. 2015. The Healing Journey: Help Seeking for Self-Injury Among a Community Population. *Qualitative Health Research,* 25**,** 932-944.

MACKENZIE, J. C., CARTWRIGHT, T. & BORRILL, J. 2018. Exploring suicidal behaviours by probation clients-a qualitative near-lethal study. *Journal of Public Health,* 40**,** 146-153.

MCANDREW, S. & WARNE, T. 2014. Hearing the voices of young people who self-harm: Implications for service providers. *International Journal of Mental Health Nursing,* 23**,** 570-579.

MCGILL, K., HACKNEY, S. & SKEHAN, J. 2019. Information needs of people after a suicide attempt: A thematic analysis. *Patient Education and Counseling*.

PARKES, J. H. & FRESHWATER, D. S. 2012. The journey from despair to hope: an exploration of the phenomenon of psychological distress in women residing in British secure mental health services. *Journal of Psychiatric and Mental Health Nursing,* 19**,** 618-628.

PAVULANS, K. S., BOLMSJO, I., EDBERG, A.-K. & OJEHAGEN, A. 2012. Being in want of control: Experiences of being on the road to, and making, a suicide attempt. *International Journal of Qualitative Studies on Health and Well-being Vol 7 2012, ArtID 16228,* 7.

PERSEIUS, K.-I., ÖJEHAGEN, A., EKDAHL, S., ÅSBERG, M. & SAMUELSSON, M. 2003. Treatment of suicidal and deliberate self-harming patients with borderline personality disorder using dialectical behavioral therapy: the patients’ and the therapists’ perceptions. *Archives of Psychiatric Nursing,* 17**,** 218-227.

READING, L. & BOWEN, E. 2014. A thematic analysis of how prisoners overcome suicidality. *International journal of prison health.,* 10**,** 212-27.

RISSANEN, M.-L., KYLMÄ, J. & LAUKKANEN, E. 2009. Descriptions of Help by Finnish Adolescents Who Self-Mutilate. *Journal of Child and Adolescent Psychiatric Nursing,* 22**,** 7-15.

RISSANEN, M. L., KYLMA, J., HINTIKKA, J., HONKALAMPI, K., TOLMUNEN, T. & LAUKKANEN, E. 2013. Factors helping adolescents to stop self-cutting: descriptions of 347 adolescents aged 13-18 years. *Journal of Clinical Nursing,* 22**,** 2011-9.

SEKO, Y., KIDD, S. A., WILJER, D. & MCKENZIE, K. J. 2015. On the Creative Edge: Exploring Motivations for Creating Non-Suicidal Self-Injury Content Online. *Qual Health Res,* 25**,** 1334-46.

SHAW, S. N. 2006. Certainty, Revision, and Ambivalence: A Qualitative Investigation into Women's Journeys to Stop Self-Injuring. *Women & Therapy,* 29**,** 153-177.

SIEGEL, K. & MEYER, I. H. 1999. Hope and resilience in suicide ideation and behavior of gay and bisexual men following notification of HIV infection. *AIDS Education & Prevention,* 11**,** 53-64.

SINCLAIR, J. & GREEN, J. 2005. Understanding resolution of deliberate self harm: qualitative interview study of patients' experiences. *BMJ,* 330**,** 1112.

SUN, F.-K., LONG, A., TSAO, L.-I. & HUANG, H.-M. 2014. The Healing Process Following a Suicide Attempt: Context and Intervening Conditions. *Archives of Psychiatric Nursing,* 28**,** 55-61.

SUN, F. K. & LONG, A. 2013. A suicidal recovery theory to guide individuals on their healing and recovering process following a suicide attempt. *Journal of Advanced Nursing,* 69**,** 2030-40.

SUTHERLAND, O., DAWCZYK, A., DE LEON, K., CRIPPS, J. & LEWIS, S. P. 2014. Self-compassion in online accounts of nonsuicidal self-injury: An interpretive phenomenological analysis. *Counselling Psychology Quarterly,* 27**,** 409-433.

TOFTHAGEN, R., TALSETH, A. G. & FAGERSTROM, L. M. 2017. Former patients' experiences of recovery from self-harm as an individual, prolonged learning process: a phenomenological hermeneutical study. *Journal of Advanced Nursing,* 73**,** 2306-2317.

VATNE, M. & NADEN, D. 2016. Crucial resources to strengthen the desire to live: Experiences of suicidal patients. *Nursing Ethics,* 23**,** 294-307.

VATNE, M. & NADEN, D. 2018. Experiences that inspire hope: Perspectives of suicidal patients. *Nursing Ethics,* 25**,** 444-457.

WADMAN, R., ARMSTRONG, M., CLARKE, D., HARROE, C., MAJUMDER, P., SAYAL, K., VOSTANIS, P. & TOWNSEND, E. 2018. Experience of Self-Harm and Its Treatment in Looked-After Young People: An Interpretative Phenomenological Analysis. *Archives of Suicide Research,* 22**,** 365-379.

WEBER, M. T. 2002. Triggers for self-abuse: a qualitative study. *Archives of Psychiatric Nursing,* 16**,** 118-24.

WHITLOCK, J., PRUSSIEN, K. & PIETRUSZA, C. 2015. Predictors of self-injury cessation and subsequent psychological growth: Results of a probability sample survey of students in eight universities and colleges. *Child and Adolescent Psychiatry and Mental Health,* 9 (1) (no pagination).

WILLIAMS, A. J., NIELSEN, E., AND COULSON, NEIL. S 2018. "They aren't all like that": Perceptions of clinical services, as told by self-harm online communities. *Journal of Health Psychology,* 1-14.

WILLS, K. A. & HONS, D. 2013. What does recovery mean to adults who self-injure? an interpretative phenomenological analysis. *International Journal of Psychosocial Rehabilitation* 17**,** 93-116.

ZAHEER, J., SHERA, W., SING HONG LAM, J., FUNG, W. L. A., LAW, S. & LINKS, P. S. 2018. "I think i am worth it. I can give up committing suicide": Pathways to recovery for chinese-canadian women with a history of suicidal behaviour. *Transcultural Psychiatry***,** 1363461518818276.
